# Volumetric modulated arc radiation therapy in the treatment of lung cancer: images of treatment planning case

**DOI:** 10.11604/pamj.2014.18.276.4108

**Published:** 2014-08-04

**Authors:** Khalid Andaloussi-Saghir, Hamid Mansouri

**Affiliations:** 1 Department of Radiotherapy Oncology, Mohammed V Military Hospital, Rabat, Morocco

**Keywords:** Arc radiation therapy, lung cancer, treatment

## Image in medicine

A 56-year-old Arabic man, with a 30 pack-year (1pack/day for 30 years) smoking history, admitted to the department of radiation oncology of military teaching hospital of Rabat for management of locally advanced non small cell cancer of the left lung (Adenocarcinoma). Computerized tomography (CT) of the chest showed a mass involving left upper lobe,measuring 6x7 cm in diameter and infiltrating the vertebral body. Workup including brain MRI and PET-CT, doesn't found lymphadenopathy in the mediastina or distant metastasis, the tumor was hyper metabolic (A). The disease was staged T4N0M0. A decision was made to proceed with chemo radiotherapy with curative aim, at the dose of 66 Gy, in 33 fractions of 2Gy, five fractions per week. The proximity of the target volume to the spinal cord,whose dose tolerance should not exceed 45 Gy, makes it impossible to achieve this radiation therapy with conventional technique using simple beams even with 3D conformal method. A treatment by volumetric modulated arc radiation therapy (Rapid arc) was initiated, and the use of two arcs allowed the optimal coverage of the target volume by isodoseline 95% without exceeding the permissible level dose in the spinal cord (B, C). Through this case, we aim to encourage acquisition of innovating technologies like Rapidarc by developing countries especially in Africa.

**Figure 1:**
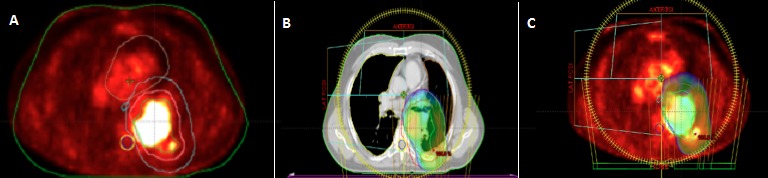
(A): target volume delineation in PET-CT; (B) et (C): coverage of the target volume by isodose line 95% (in blue)

